# Research on Toughening and Damping Application of Epoxy Resin-Based Carbon Fiber-Reinforced Composite Material

**DOI:** 10.3390/ma19040815

**Published:** 2026-02-20

**Authors:** Wei Wang, Xueping Gao, Zhimin Li, Yishi Wang, Bo Zhu

**Affiliations:** 1 Carbon Fiber Engineering Research Center, School of Materials Science and Engineering, Shandong University, Jinan 250061, China; wangwei2023@mail.sdu.edu.cn (W.W.); lizhimin2023@mail.sdu.edu.cn (Z.L.); yswang88@126.com (Y.W.); 2 Key Laboratory of Liquid-Solid Structural Evolution and Processing of Materials of Ministry of Education, Shandong University, Jinan 250061, China

**Keywords:** epoxy resin, carbon fiber, carboxyl-terminated butadiene rubber, toughening, damping

## Abstract

Carbon fiber-reinforced resin matrix composites (CFRC) are extensively used in aerospace, automotive manufacturing, and sports equipment. However, the brittle nature of the resin matrix causes CFRC to exhibit severe vibrations and noise under dry friction conditions. Enhancing the intrinsic damping properties of the resin matrix serves as a fundamental and effective strategy to mitigate vibration and noise radiation in composite components. This study systematically investigates high-temperature co-curing damping composites using co-curing technology, aiming to improve the mechanical performance and damping characteristics of traditional fiber-reinforced epoxy resin composites. A novel carbon fiber-reinforced terminal carboxyl nitrile epoxy pre-polymer composite material demonstrates both stable chemical properties and excellent high-temperature resistance. Through formulation adjustments, the curing temperature and time of epoxy resin are matched with those of the terminal carboxyl nitrile epoxy pre-polymer. The performance of epoxy carbon fiber composites was evaluated through tensile tests, flexural tests, impact tests, infrared spectroscopy, thermogravimetric analysis, dynamic mechanical analysis, scanning electron microscopy, and X-ray diffraction. Results show that blending epoxy resin with terminal carboxyl nitrile liquid rubber enhances energy dissipation by increasing intermolecular friction and hydrogen bonding interactions. The damping ratio of epoxy resin-based carbon fiber composites reaches as high as 1.67%. Tensile strength, flexural strength, and impact strength reach 1968 MPa, 1343 MPa, and 127 kJ/m^2^, respectively. The addition of terminal carboxylated nitrile liquid rubber facilitates the formation of continuous friction membranes, enhancing friction stability. Tensile tests demonstrate that carbon fiber composites containing 25% terminal carboxylated nitrile liquid rubber outperforms other formulations. As evidenced by impact tests, the performance of the prepared composites is superior to that of other configurations. Dynamic mechanical analysis indicates that the 25% rubber-containing composites exhibit enhanced damping characteristics and higher loss modulus. Experimental results confirm that this study advances the development of functional composites for vibration reduction and noise control applications.

## 1. Introduction

The rapid advancement of science and technology has significantly accelerated the automation of mechanical equipment. However, this progress has been accompanied by issues of vibration and noise, which not only contribute to environmental pollution but also pose risks to people’s physical and mental health. Damping materials, which convert mechanical energy from external impacts and vibrations into heat for dissipation, offer an effective solution to these problems. Among various types, polymer damping materials are widely used due to their ease of processing and molding, structural design flexibility, low cost, and unique viscoelastic properties. Nevertheless, during practical application, prolonged exposure to external loads can lead to the formation and propagation of micro-cracks and damage in these materials, eventually resulting in complete failure and rendering them unusable [1,2,3]. High-damping materials are capable of effectively mitigating pulse impact loads, which in turn diminishes the propensity for brittle fracture and prolongs the service life of structural components. Owing to their desirable characteristics—such as low density, high strength, high stiffness, and corrosion resistance—the development of high-damping carbon fiber composites has become a major focus among researchers [4].

Epoxy resin is a thermosetting polymer material known for its excellent adhesion, corrosion resistance, electrical insulation, and high strength [5]. Due to these properties, it is widely employed as a matrix resin in composite materials and finds extensive application in various manufacturing sectors, including aerospace, automotive, wind power, nuclear power, construction, and shipbuilding [6,7,8,9,10,11,12]. The resin matrix consists of various epoxy resins, curing agents, accelerators, and modifiers. Epoxy resins must react with curing agents to form a three-dimensional cross-linked network, enabling them to meet diverse molding processes and performance requirements. This highly cross-linked structure enhances the mechanical and thermal properties of the cured epoxy resin. However, excessive cross-linking density and a rigid covalent network can result in poor impact resistance and insufficient toughness. Based on this, fiber materials are commonly used to enhance the damping performance of epoxy resin composites. This interfacial layer possesses properties distinct from both the fiber and the matrix, as the internal friction between fibers and resin plays a crucial role in the overall performance of the composite material [13]. However, the brittleness of the resin matrix makes these composites prone to severe vibration and noise under dry friction conditions. If the fracture elongation of the resin matrix is too low, it cannot meet the requirements for winding processes, inevitably limiting the future development and application of epoxy resin as a high-performance composite material. Consequently, toughening modification of epoxy resin has become a highly active area of research [14]. Fei et al. [15,16] investigated the toughening effect of tannic acid derivatives on epoxy resin. They utilized renewable tannin to synthesize dodecyl-functionalized tannin, which was incorporated into an epoxy/acid anhydride system. The toughening effect produced by this modification while maintaining the tensile strength, modulus, and glass transition temperature of the material is not significant. Therefore, the preference for synthetic fibers such as carbon fiber over natural fibers stems primarily from their superior strength-to-weight ratio, rigidity, durability, and resistance to moisture and corrosion properties that are essential in high-performance applications like automotive and aerospace industries [17]. For example, Li et al. [18] investigated the tensile strength of carbon fiber laminates modified with EFPS/nano-SiO_2_. The results indicated that the epoxy resin enhanced the transfer of external stress to the carbon fiber more effectively than pure epoxy resin. Zhu et al. [19] investigated the drilling process of carbon fiber-reinforced plastics (CFRP). They proposed a method to optimize the drilling process, which reduced drilling-induced defects, improved hole quality, and provided a valuable reference for the machining of composite materials. Isik et al. [20] used unidirectional glass fiber to fabricate composites (UD-GFRP) and processed the material. They recommended using optimal cutting parameters to achieve superior surface quality. Robinson et al. [21] developed a perforated damping model to optimize both the stiffness and the damping loss factor of laminated plates. The experimental results were consistent with the finite-element simulations.

This study investigates closed-loop damping in the epoxy resin–prepreg–composite system. Based on E51 bisphenol an epoxy resin, carboxyl-terminated nitrile liquid rubber (CTBN)—a mature material in toughening and damping applications—was selected and epoxy-modified to produce carboxyl-terminated nitrile epoxy pre-polymers (EPBN). EPBN was incorporated into the epoxy matrix at varying loadings of 5%, 15%, 25%, and 35%. Based on this, carbon fiber-reinforced polymer (CFRP) was blended to prepare epoxy resin-based carbon fiber composites, designated as CF-EP5%, CF-EP15%, CF-EP25%, and CF-EP35% throughout the article. Mechanical properties and damping performance tests were conducted on the epoxy resin-based carbon fiber composites. Morphological characterization and evaluation of mechanical properties and damping performance were performed using techniques including Fourier Transform Infrared Spectroscopy (FT-IR), Thermogravimetric Analysis (TG), and Dynamic Mechanical Analysis (DMA). This research involves chemically grafting CTBN onto the E51 epoxy resin backbone to synthesize structurally defined EPBN and systematically applying it to the toughening and damping field of CFRP, thereby achieving chemical bonding between the rubber phase and the epoxy resin, compared to traditional physical blending. This ensures a more uniform phase separation structure and superior interfacial stability. This study provides novel design concepts and experimental evidence for the preparation of advanced composite materials with damping properties, high toughness, and high strength.

## 2. Materials and Methods

### 2.1. Materials

Epoxy resin 188EL (epoxy equivalent: 182–192; viscosity at 25 °C: 1000–15,000 cps) was supplied by Qingdao Zhongke Composite Materials Co., Ltd., Qingdao, China. T700 12K Carbon fiber was obtained from Weihai Guangwei Group Co., Ltd., Weihai, Shandong, China. The curing agent H106 and catalyst F401 (particle size: d50 ≤ 5 μm; melting point: 180–195 °C; volatile content ≤ 1%) were obtained from Changzhou Calla Resin Co., Ltd., Jiangsu Province, China. Liquid carboxyl-terminated butadiene acrylonitrile rubber (CTBN) with a carboxyl value of 0.51–0.65 mol/g, acrylonitrile content of 22.1–27%, and volatile content ≤ 2.0% was sourced from Tianyuan Aviation Materials (Yingkou) Technology Co., Ltd., Liaoning, China.

### 2.2. Equipment and Instruments

DJ-502 electronic balance (Fuzhou Huazhi Scientific Instrument Co., Ltd., Fuzhou, China);

Q500 thermal gravimetric analyzer (TA Instruments, Shanghai, China);

Vacuum oven (Jinan Shangdi Electronic Technology Co., Ltd., Jinan, China);

U4503 electronic universal testing machine (Shenzhen Sanyishengtong Technology Co., Ltd., Shenzhen, China);

DMA dynamic mechanical analyzer (Mettler-Toledo, Changzhou, China);

DRB-215A AutoFlow waterjet (Nanjing Xunrun Machinery Technology Co., Ltd., Nanjing, China);

DHR rheometer (TA Instruments, Shanghai, China);

JB-160 electric stirrer (Xiniu Technology Co., Ltd., Hangzhou, China);

Scanning Electron Microscope (Regulus 8100) (purchased from Hitachi, Suzhou, China);

X-ray diffractometer (Smart Lab 3KW) (purchased from Rigaku of Japan, Tokyo, Japan);

PCD-40S Hot Pressing Machine (MAIKE, Dongguan, China);

Ten-ton Universal Tensile testing machine/501J Pendulum Impact Tester (purchased from Shenzhen Wantech Testing Equipment Co., Ltd., Shenzhen, China).
**Device name****Device model****Device manufacturers**Microcontroller digital display heating plateEH20DBeijing LabTech Instrument Co., Ltd., Beijing, ChinaElectronic balanceDJ-502Fuzhou Huazhi Scientific Instrument Co., Ltd.Vacuum oven HG-420Jinan Shangdi Electronic Technology Co., Ltd. Electronic universal testing machineU4503Shenzhen Sanyishengtong Technology Co., Ltd.Dynamic mechanical analyzerDMA-861eMETTLER TOLEDO Technology (China) Co., Ltd.AutoFlow waterjetDRB-215ANanjing Xunrun Machinery Technology Co., Ltd.RheometerDiscovery HR-2TA Instruments Waters Technology (Shanghai) Co., Ltd.Electric stirrerJB-160Xiniu Technology Co., Ltd.Scanning Electron Microscope Regulus 8100Hitachi Instruments (Suzhou) Co., Ltd.X-ray diffractometersSmart Lab 3KWJapan Rigaku Co., Ltd.Hot Pressing molding MachinePCD-40S Maike Electronic Technology Co., Ltd. Universal Tensile testing machine10T Shenzhen Wantech Testing Equipment Co., Ltd.Pendulum Impact Tester 501J Shenzhen Wantech Testing Equipment Co., Ltd.

### 2.3. Preparation of Damping Materials

This study focuses on the closed-loop damping behavior of an epoxy resin–prepreg–composite system. The fiber-reinforced composite laminates were fabricated using unidirectional prepreg with an epoxy resin volume fraction of 40%. Based on E51 bisphenol a epoxy resin, carboxyl-terminated butadiene acrylonitrile liquid rubber (CTBN)—a widely used material in the field of toughening and damping—was selected and epoxidized to obtain an epoxy-functionalized carboxylated butadiene acrylonitrile pre-polymer (EPBN). Subsequently, EPBN was blended with carbon fiber (CFRP) to prepare epoxy resin-based carbon fiber composites. An experimental formulation was developed based on the curing process of epoxy resin and the content of curing agents. By varying the curing agent content and measuring the properties of different epoxy resin formulations, the optimal formulation was determined. The curing temperature and time for the epoxy resin were set at 120 °C and 150 min, respectively. We prepared the optimal E51 bisphenol-a epoxy resin system based on the best formulation. In a beaker, to the E51 bisphenol-a epoxy resin system, a specific amount of curing agent and accelerator was added and stirred with an electric mixer and then cured to obtain the optimal casting system. The stirring speed was 200 rpm, and the stirring temperature was 60 °C during the preparation of the curing agent and resin.

**Figure 1:**
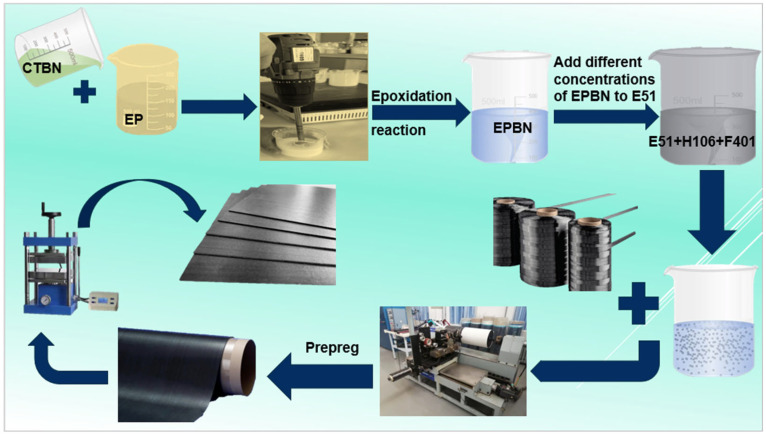
Flowchart of the EPBN-Modified Epoxy Resin and Carbon Fiber Composite Fabrication Process.

E51 bisphenol-a epoxy resin and EPBN (5%, 15%, 25%, and 35% by weight) were blended at specified ratios. Subsequently, the curing agent was added to the resin solution at a mass ratio of 1:3, followed by mechanical stirring for 120 min. The mixture was then placed in a vacuum drying oven to remove air bubbles. Unidirectional carbon fiber prepregs were fabricated by thoroughly impregnating carbon fibers with the EPBN-modified epoxy resin system using an automated fiber placement and impregnation machine. The prepreg exhibited a fiber areal density of 100 g/m^2^ and an epoxy resin content of 40%. The prepreg was cut to the required size, and twenty layers were stacked in the specified orientation. A composite plate was then fabricated using press molding (1.5 MPa), resulting in a material thickness of 2 and 4 mm. The curing process was carried out at 120 °C for 150 min, with the carbon volume fraction in the cured laminate controlled at 60%. A heating rate of 3 °C/min and a cooling rate of 2 °C/min were applied. The slow cooling rate was implemented to minimize the effects of residual stress [22]. The experimental flowchart is shown in Figure 1. Furthermore, concerning fiber orientation, unidirectional T700 12K carbon fiber was used for the carbon fiber, and unidirectional UD prepreg was prepared by unidirectional pre-impregnation. The laminated board was prepared by laminating and molding at the same angle and direction.

### 2.4. Characterization

1. Determination of mechanical properties of composite films:
(1) Tensile tests were performed on epoxy resin-based carbon fiber-reinforced composites in accordance with ISO 527-5:2021 [23]. Testing was conducted using a U4503 electronic universal testing machine equipped with a 10 kN load cell. All tests were carried out with a constant crosshead speed of 20 mm/min. The specimen dimensions were 230 mm × 15 mm × 2 mm;
(2) Flexural properties were determined according to ISO 14125:1998 [24]. A three-point bending test was performed at a loading rate of 2 mm/min using a dedicated flexural fixture (Flex Sensor 4.5) with a support span of 64 mm. The specimen dimensions for this test were 80 mm × 12.5 mm × 2 mm. The flexural strength is

(1) $$\sigma_{f}=\frac{3PL}{2bh^{2}}$$

In the formula, σ_f_ is the flexural strength (MPa).

P is the load applied to the flexure specimen (*N*).

*L* the span (mm).

*b* is the width (mm).

*h* for thickness (mm).

The formula for calculating the flexure modulus is as follows:

(2) $$E=\frac{L^{3}K}{4bd^{3}}$$

where *E* is the modulus of elasticity (GPa).

*L* is the span (mm).

*K* is the tangent of the initial straight part of the curve (N/mm).

*b* is the width of the sample (mm).

*d* is the thickness of the sample (mm);

(3) Impact strength was evaluated following ISO 179-1:2023 [25]. Tests were conducted using a pendulum impact tester with a 7.5 J hammer. The specimen dimensions were 80 mm × 10 mm × 4 mm.
2. FT-IR: Fourier transform infrared spectroscopy was used to scan 32 times with a resolution of 4 cm^−1^ to measure the infrared spectra of the sample in the range of 4000–400 cm^−1^.
3. TG was employed to assess the thermal stability of the composite materials, according to the ISO 11358-1:2022 [26] standard test method for sample thermal stability. Approximately 10 mg of each sample was heated from ambient temperature to 800 °C at a heating rate of 20 °C/min under a nitrogen atmosphere with a flow rate of 10 mL/min.
4. DMA: Dynamic mechanical properties were measured using a dynamic mechanical analyzer in accordance with ISO 6721-5:2019 [27]. Tests were performed in three-point bending mode with a strain amplitude of 1%. Temperature scans were conducted from 30 °C to 260 °C at a heating rate of 5 K/min under a constant frequency of 1 Hz. The specimen dimensions for DMA were 50 mm × 10 mm × 2 mm.

Furthermore, all mechanical performance data and DMA test results were obtained from specimens in the 0° fiber direction. Prior to testing, all specimens were conditioned under a standard laboratory atmosphere (23 ± 2 °C and 50 ± 10% relative humidity) for a minimum of 88 h in accordance with ISO 291:2008 [28]. Four specimens were used for each test. The data presented in this article are the average values.

5. SEM: A scanning electron microscope (Regulus 8100) was purchased from Hitachi of Japan.
6. XRD: The composite film was cut into 2.0 cm squares and tested on a Smart Lab 3 KW X-ray diffractometer at a scanning speed of 10°/min and a scanning angle of 5–80°.

## 3. Results

### 3.1. Mechanical Properties

**Figure 2:**
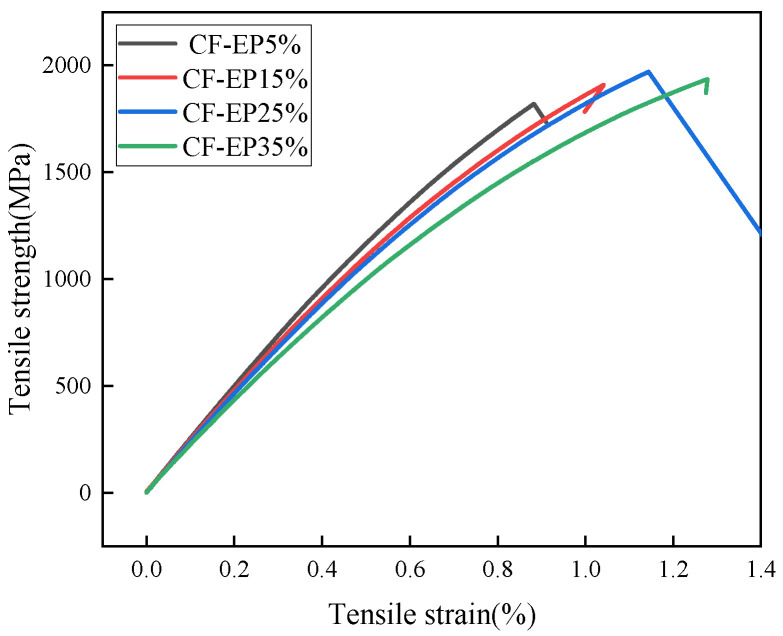
Tensile Strength of Carbon Fiber Composites with Different EPBN Content.

**Figure 3:**
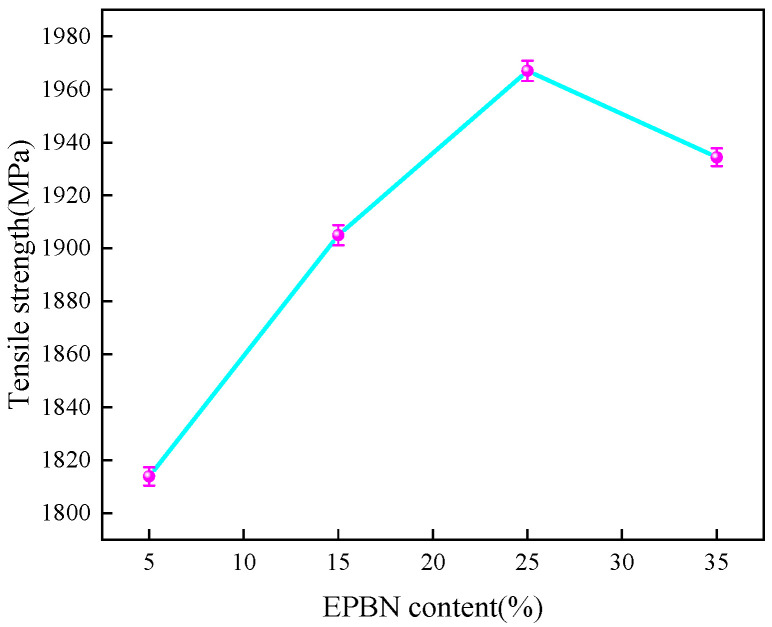
Tensile Strength of Carbon Fiber Composites with Different EPBN Content.

As shown in Figure 2, the trend before stress reaches its peak changes as the EPBN content ranges from 5% to 35%. With increasing content, the stress–strain curve exhibits an initial rise followed by a decline, and the peak stress may vary. This reflects changes in the material’s tensile strength and strain-hardening capacity. The stress–strain curve indicates that the initial slope of the curve varies little across different EPBN addition levels, suggesting that the elastic modulus is minimally affected by EPBN content. The initial slopes of the EPBN addition curves show little variation, indicating that the elastic modulus is minimally affected by EPBN content—the tensile modulus peaks at 120 GPa when CF-EP is 5%. As shown in Figure 2 and Figure 3, the tensile strength of the epoxy resin-based carbon fiber composite material first increases and then decreases with increasing EPBN content. The tensile strength reaches its maximum at 25% content, peaking at 1968 MPa. When CF-EP reaches 5%, the tensile strength achieves its minimum value of 1813 MPa. Table 1 indicates that as the EPBN content increases, the maximum force increases gradually, the tensile modulus decreases gradually, and the strain increases gradually. This demonstrates that EPBN addition enhances the material’s mechanical properties, including tensile strength and toughness. Annadorai E. M. et al. [29] investigated three distinct fiber orientations in laminates, namely [0°], [0°/90°], and [0°/90°/45°/−45°], and conducted tests on two different thicknesses (2.4 mm and 3.6 mm). Results indicated that unidirectional fiber orientation [0°], where fibers align parallel to the loading direction, achieves high tensile and flexural strengths, with maximum tensile strength reaching 756.71 MPa and maximum flexural strength at 591.45 MPa. The present study yields quite favorable results compared to previous research. Moreover, the tensile strength of CF-EP composites with different EP contents all increases rapidly to a peak and then decreases with the increase in tensile strain (deformation value), exhibiting a typical tensile stress–strain characteristic. With the EP content rising from 5% to 35%, the deformation value corresponding to the maximum tensile strength of the material first increases and then decreases; the CF-EP15% and CF-EP25% samples show a higher deformation value at the peak strength, and the ultimate deformation capacity gradually improves with the increase in EP content (the fracture strain of CF-EP35% is the largest, while that of CF-EP5% is the smallest). Among them, the material with an EP content of 15%~25% has both a high peak strength and good deformation capacity, reflecting the synergistic regulatory effect of EP content on the deformation and strength of the composite.

**Figure 4:**
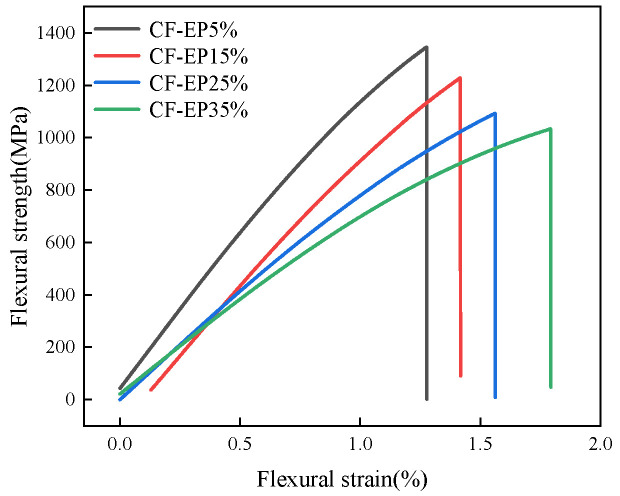
Flexural Stress–Strain of Carbon Fiber Composites with Different EPBN Content.

**Figure 5:**
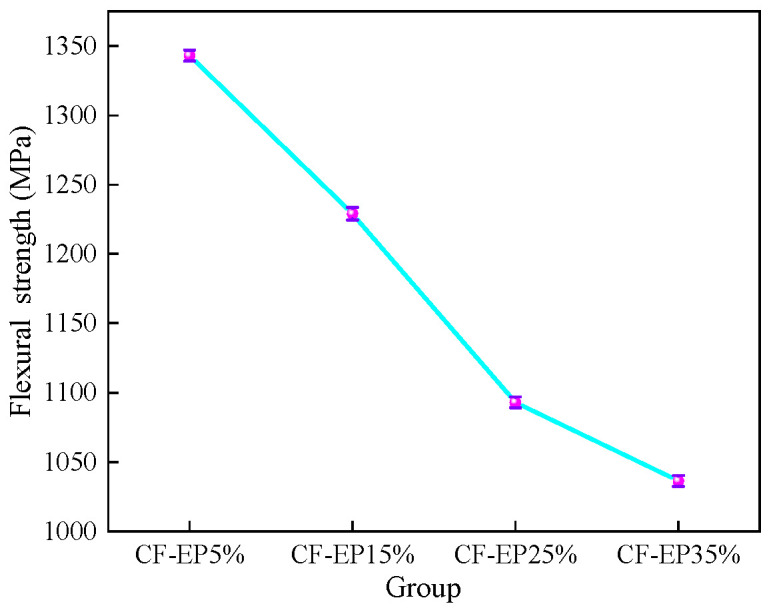
Flexural Strength of Carbon Fiber Composites with Different EPBN Content.

**Figure 6:**
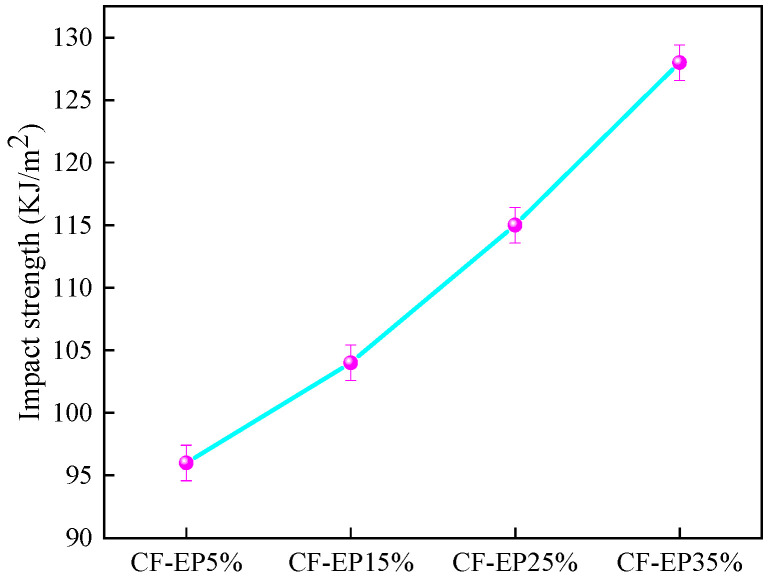
Impact Strength of Carbon Fiber Composites with Different EPBN Content.

As shown in Figure 4 and Figure 5 and Table 2, the stress–strain curves under different EPBN addition levels reflect variations in flexural strength. As the EPBN content increases, the flexural strength exhibits a changing trend; within a certain strain range, the amount influences the material’s ability to withstand flexural stress. At 5% CF-EP content, flexural strength reaches 1343 MPa, and flexural modulus reaches 118 GPa. At 35% CF-EP content, flexural strength reaches 1037 MPa, and flexural modulus reaches 100 GPa. One possible explanation is that higher toughness corresponds to reduced stiffness, making the material more prone to flexural stress. This indicates that both flexural strength and modulus are affected by the amount of EPBN added. The values of flexural strength, flexural modulus, and flexural strain vary with different EPBN addition levels. Dong et al. [30] prepared a high-damping carbon fiber/epoxy (CF/EP) composite reinforced with microcapsule particles. The effects of different microcapsule compositions on the mechanical and damping properties of the CF/EP composite were investigated. The study revealed that when the microcapsule content reached 3 vol%, the flexural strength of the CF/EP composite increased by 1.68%, achieving 734.392 MPa. In contrast to the toughening strategy employing EPBN in this study, the microcapsule modification reported in the literature focuses more on maintaining or marginally enhancing stiffness and strength through the dispersion of rigid particles. The observed trends in property changes—where EPBN leads to a decrease in strength while microcapsules result in a slight increase—are both consistent with the inherent characteristics of their respective modifiers. The significantly higher absolute flexural strength values obtained in this work (1037–1343 MPa) compared to the literature value (~734 MPa) are primarily attributed to the superior baseline performance of the carbon fiber/epoxy matrix system employed in the present study. This underscores the divergent outcomes arising from different material systems and modification objectives. As shown in Figure 6, the impact strength initially increases as the EPBN content rises. When CF-EP reaches 35%, the impact strength achieves 127 kJ/m^2^. This enhancement can be attributed to the toughening mechanism of the dispersed rubber phase. The EPBN particles act as stress concentrators, promoting localized plastic deformation, crazing, and shear banding within the epoxy matrix, which dissipates a substantial amount of impact energy. Higher impact strength indicates that the material can absorb more energy upon impact and exhibits greater plastic deformation capacity before fracture, reflecting better toughness. Conversely, lower impact strength suggests reduced energy absorption and less plastic deformation before failure, indicating relatively poor toughness.

### 3.2. Fourier Transform Infrared Spectroscopy

**Figure 7:**
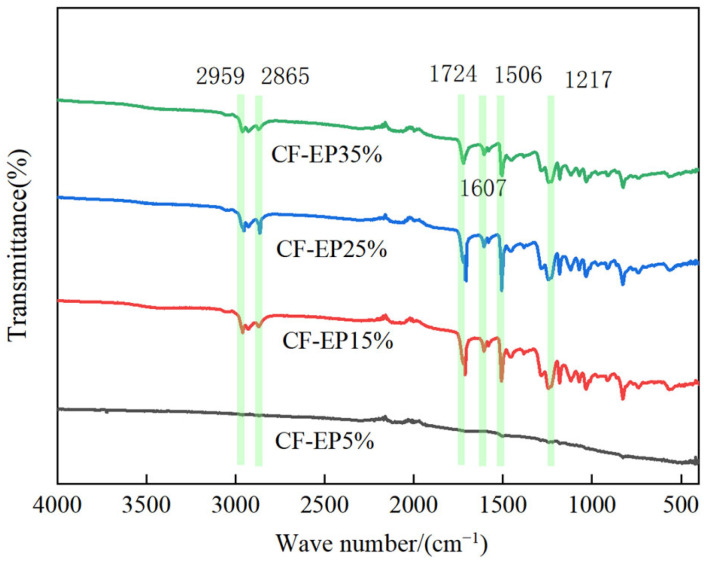
FT-IR analysis of Carbon Fiber Composites with Different EPBN Content.

As shown in Figure 7, the absence of a prominent absorption peak at 3500–3700 cm^−1^ indicates that the hydroxyl groups in the liquid acrylonitrile-based polymer largely participate in the reaction. An asymmetric stretching vibration of the unsaturated C-H bond is observed at 2959 cm^−1^, indicating that the benzene ring structure in the epoxy resin exhibits absorption in this region within the epoxy resin and carbon fiber composite system. At 2865 cm^−1^, a stretching vibration absorption peak of saturated C-H bonds appears, indicating the presence of saturated alkyl structures in the system, where the terminal carboxyl group nitrile liquid adhesive crosslinks with the epoxy resin. At 1724 cm^−1^, an absorption corresponding to the stretching vibration of the C=O bond appears, likely arising from the reaction between the epoxy resin and the liquid end-capped carboxyl nitrile rubber. At 1607 cm^−1^, absorption of the C=C bond stretching vibration is observed, indicating the formation of conjugated double bonds during the reaction. At 1506 cm^−1^, absorption of the benzene ring backbone vibration is observed, indicating the presence of benzene ring functional groups in the epoxy resin structure. Additionally, the C-O bond stretching vibrations appear at 1217 cm^−1^, which may correspond to absorption peaks from functional groups such as ether bonds, alcohol hydroxyl groups, or phenol hydroxyl groups in the epoxy resin. Modified epoxy resins with varying concentrations of carboxylic acid-terminated nitrile liquid rubber (CF-EP5%, CF-EP15%, CF-EP25%, and CF-EP35%) may cause changes in the intensity and position of absorption peaks for different functional groups. By comparing these changes, we can further analyze the impact of different modification levels on the chemical structure of the system.

### 3.3. Thermogravimetric Analysis

**Figure 8:**
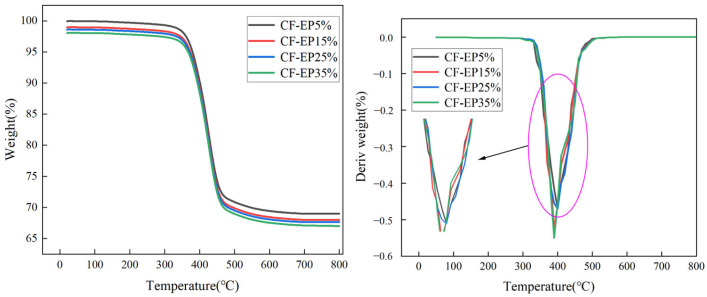
TG analysis of Carbon Fiber Composites with Different EPBN Content.

As shown in Figure 8, three distinct stages of thermal degradation can be observed. The changes in mass are shown in Table 3. The primary quality loss in the first stage stems from the evaporation of CTBN, curing agents, accelerators, and moisture added during the preparation of EPBN. CTBN exhibits lower thermal stability than epoxy resin and undergoes rapid thermal decomposition. The primary cause of mass loss in the second stage is thermal degradation reactions triggered by the unsaturated bonds and polar groups present in the acrylonitrile rubber fraction of undecomposed CTBN, resulting in significant mass loss. The primary quality loss in the third stage stems from epoxy resin that was not fully decomposed during the second stage, which continues to undergo pyrolysis reactions at this higher temperature range. Ultimately, the mass loss rate stabilizes due to the high-temperature tolerance of carbon fiber.

The initial thermal decomposition temperature (the point at which significant weight loss begins), the rate of weight loss, and the final residual mass vary with different amounts of EPBN addition. Generally, a higher initial decomposition temperature, a slower weight loss rate, and a greater residual mass indicate better thermal stability of the material. As the EPBN addition increases from 5% to 35%, differences in the initial thermal decomposition temperature are observed. At lower addition amounts, the initial decomposition temperature is relatively higher, suggesting that the material is more resistant to the onset of thermal decomposition and exhibits better thermal stability. Furthermore, different EPBN contents also affect both the rate and the extent of weight loss. The slope of the curve reflects the rate of weight loss. A steeper slope is observed at 5% EPBN addition, indicating a faster weight loss rate, while a gentler slope at 25%EPBN addition corresponds to a slower weight loss rate. These results demonstrate that the EPBN content significantly influences the thermal stability of epoxy resin-based carbon fiber-reinforced composites.

### 3.4. Dynamic Mechanical Analysis

**Figure 9:**
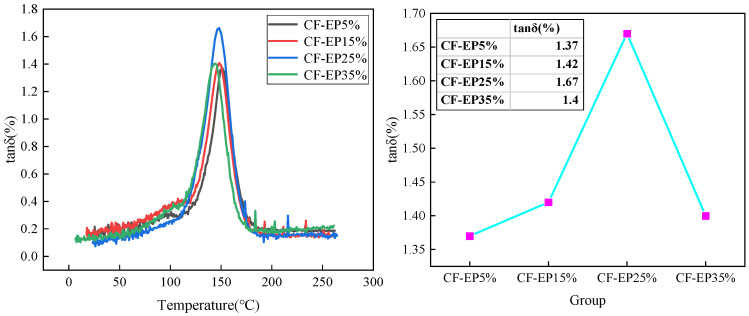
Temperature-dependent damping ratio curves for carbon fiber materials with different EPBN contents.

Figure 9 presents the DMA curves of epoxy resin-based carbon fiber-reinforced damping and toughening materials with different EPBN contents. In the lower-temperature region (below the glass transition), the curves show minimal variation across different EPBN additions, and the storage modulus remains relatively stable. This indicates that the EPBN content has a negligible effect on the material’s modulus in the glassy state. As the temperature increases into the transition region (approximately 110–150 °C), significant changes in the viscoelastic response become apparent. Notable differences in the peak height of tanδ% are observed among samples with different EPBN contents. The peak is most pronounced at 25% EPBN addition, indicating a higher damping capacity and more effective energy dissipation within this temperature range. Conversely, the sample with 5% EPBN addition exhibits a lower peak, corresponding to a less pronounced damping response.

**Figure 10:**
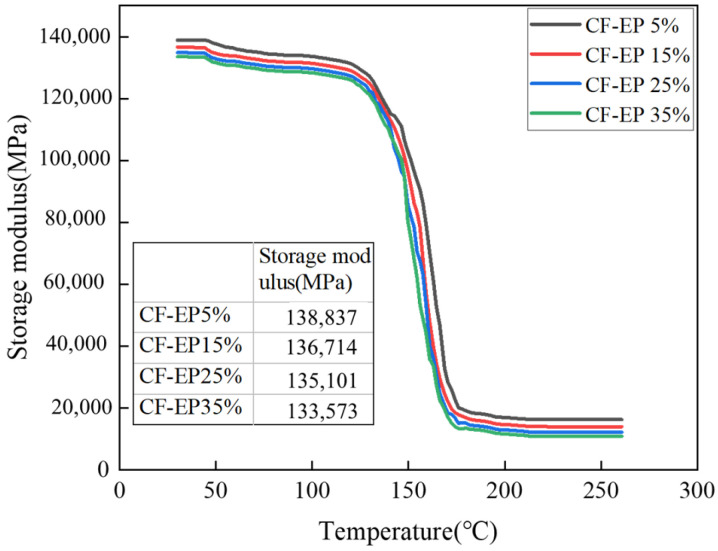
Temperature-dependent storage modulus curves for carbon fiber materials with varying EPBN content.

As shown in Figure 10, the initial storage modulus (measured in the glassy state) decreases with increasing EPBN content. Under greenhouse conditions (23 ± 2 °C, 50 ± 10% RH), the storage modulus of the CF-EP5% material is 138,837 MPa, that of the CF-EP15% material is 136,714 MPa, that of the CF-EP25% material is 135,101 MPa, and that of the CF-EP35% material is 133,573 MPa. This trend can be attributed to the reduced rigidity of the epoxy matrix upon incorporation of the EPBN modifier, as described by the rule of mixtures for composite materials, given a constant carbon fiber volume fraction. Furthermore, the EPBN content primarily influences the rate and extent of the storage modulus drop during the glass transition. A higher EPBN content generally accelerates this decrease, indicating a reduction in the material’s thermal stability and load-bearing capacity at elevated temperatures. Cai et al. [31] prepared poly (sulfon) (PSF)/cellulose nanocrystal (CNC) nanofiber membranes via electrospinning as hybrid integrated interlayers in CF/EP composites. The damping properties of six different interlayer CF/EP composites were tested. The results indicate that the damping ratio reached 1.04% for the 10%PSF/0.5% CNC–3I composite. This study concludes that the damping ratio of CF-EP25% epoxy resin-based carbon fiber composite materials reaches 1.67%, demonstrating significantly superior damping performance compared to some related studies. In general, the EPBN content has a noticeable influence on the damping performance of carbon fiber-reinforced materials.

### 3.5. SEM Analysis

**Figure 11:**
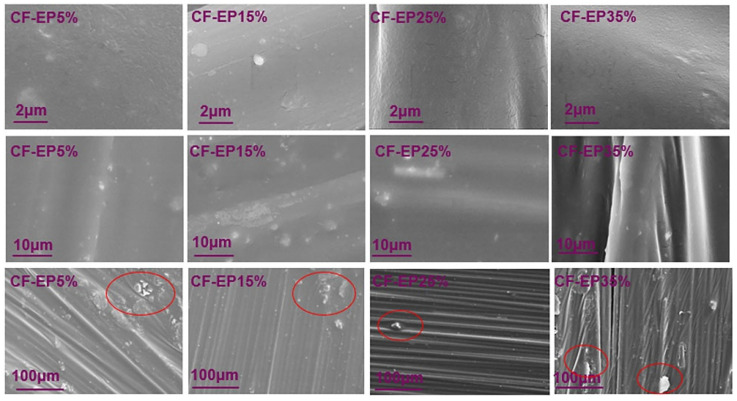
SEM analysis of Carbon Fiber Composites with Different EPBN Content.

As shown in Figure 11, surface morphology images of CF-EP materials with different carbon fiber content are compared at various magnifications. The figure clearly reveals the outlines of carbon fibers, which exhibit a relatively uniform fibrous morphology. The surface is not completely smooth, with some granular substances attached, indicating that the epoxy resin matrix is relatively uniformly coated on the carbon fiber. Overall, no obvious large pores or severe delamination are observed in the image, suggesting that there is a certain bonding strength between the carbon fiber and the epoxy resin matrix. This is beneficial to the performance of the composite material. When the CF-EP content is 5%, the amount of CF-EP adhering to the carbon fiber surface is insufficient, resulting in poor interfacial bonding. The carbon fiber contours remain clearly visible, indicating reduced toughness and a tendency toward brittle fracture. When CF-EP reaches 15%, the carbon fiber surface is observed to be uniformly coated with EPBN, exhibiting some micro-pores or traces of phase separation. This occurs because the addition of CTBN affects the cross-linking structure of EPBN, reducing the bonding strength between EPBN and carbon fibers. When CF-EP reaches 25%, the coverage of EPBN on carbon fibers significantly improves. EPBN adheres more tightly to the carbon fibers without extensive agglomeration, enhancing interfacial bonding strength. This endows the epoxy-based carbon fiber composite material with superior mechanical properties. When CF-EP35% is used, agglomeration occurs on the carbon fiber surface, and phase separation of EPBN is observed. This may be attributed to the increased brittleness of the epoxy resin–carbon fiber composite caused by the addition of CF-EP35%. The cured carbon fiber exhibits heightened brittleness, and excessive toughening agents weaken the interface, leading to pronounced agglomeration and a subsequent decline in mechanical properties. These characteristics indicate that the addition of EPBN enhances the interfacial bonding strength of the material, thereby improving its mechanical properties.

### 3.6. XRD Analysis

**Figure 12:**
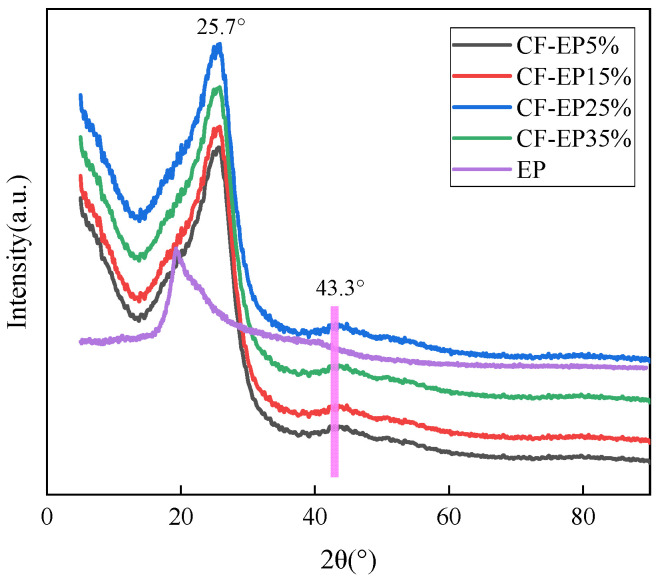
XRD analysis of Carbon Fiber Composites with Different EPBN Content.

As depicted in Figure 12, the E-P coating presents a broadened diffraction peak at 20°, which can be attributed to the crystallization generated during the curing and crosslinking process of the resin. This observation is in accordance with the findings of previous studies. Under designated curing conditions, the molecular chains of epoxy resin in the carboxyl-terminated nitrile liquid-modified epoxy resin system are capable of arranging in an orderly fashion, giving rise to local crystalline regions that generate corresponding diffraction peaks. Consequently, the pure epoxy resin displays a broad diffraction peak at 2θ = 20° [32]. The diffraction peaks of the composite material at 2θ = 25.7° and 43.3°are attributed to the (200) and (100) crystalline diffraction peak of carbon, which verify the successful compounding of carbon fibers [33,34]. Furthermore, the broadening of the diffraction peaks can be ascribed to the elevated amorphization degree of the composite material, resulting from the interfacial bonding between the amorphous resin matrix and graphite. Among the samples, CF-EP25% exhibits the most pronounced diffraction peak broadening at 2θ = 25.7°, suggesting that the carbon fiber and the amorphous resin form an excellent interfacial bond under this condition. This result is in good agreement with the conclusions derived from mechanical property tests, where a robust interfacial adhesion between the reinforcing phase and the matrix material contributes to improve the outstanding mechanical performance of the composite.

## 4. Discussion

**Figure 13:**
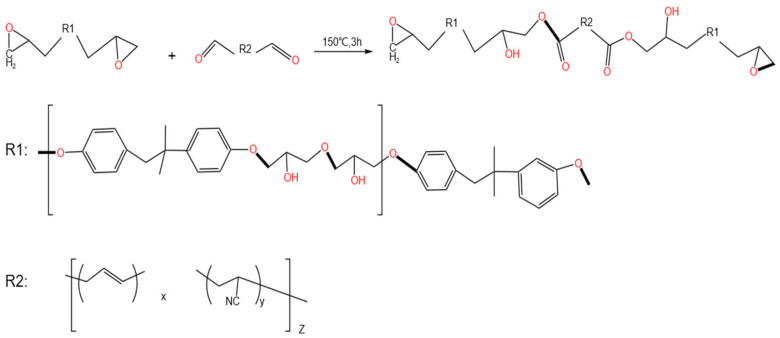
Reaction Mechanism of CTBN and EP.

The performance enhancement of CTBN-modified epoxy/carbon fiber composites stems from a multi-level, multi-scale synergistic mechanism encompassing the entire process from molecular chemical bonding and micro-phase separation to fiber-matrix interface interaction and macroscopic property regulation. The process initiates during the pre-polymer synthesis stage, where carboxyl-terminated liquid butadiene acrylonitrile rubber (CTBN) and E51 epoxy resin undergo a ring-opening esterification reaction under heating. The carboxyl groups (–COOH) of CTBN react with epoxy groups, forming ester linkages (–COO–) and producing a novel block copolymer—the carboxyl-terminated butadiene acrylonitrile epoxy pre-polymer (EPBN) [35]. This pre-polymer incorporates both rigid segments (derived from the epoxy resin) and flexible segments (originating from the CTBN rubber). Evidence for this reaction is provided by FT-IR, which reveals the characteristic C=O stretching vibration peak of the ester bond at 1724 cm^−1^, while the hydroxyl absorption peak is indistinct in the 3500–3700 cm^−1^ region [36]. During the curing process, a reaction-induced phase separation occurs. Following the addition of the curing agent, the epoxy components in EPBN participate in constructing a three-dimensional crosslinked network [37]. Meanwhile, the flexible segments of CTBN, exhibiting poor compatibility with the epoxy network structure, gradually segregate to form a dispersed rubber phase and a continuous epoxy resin phase, thereby enhancing the mechanical properties of the composite material (for the reaction equation, see Figure 13).

The carbon fiber surface exhibits a certain degree of roughness. During the curing process, the epoxy resin penetrates the uneven surface of the fiber, forming a mechanical interlocking effect that enhances the interfacial bonding performance between the carbon fiber and the resin. Additionally, an interfacial phase exists between the fiber and the epoxy resin, the structure and properties of which play a critical role in determining the overall interfacial characteristics of the composite. The thickness, chemical composition, and microstructure of this interfacial phase directly influence the efficiency of stress transfer between the carbon fiber and the epoxy matrix. An ideal interfacial phase should exhibit moderate thickness and suitable mechanical properties, allowing it to transfer stress while mitigating stress concentration effectively. In terms of interfacial bonding with carbon fibers, the EPBN pre-polymer operates through a dual mechanism: its flexible chain segments effectively wet the fiber surface, reducing interfacial defects, while residual epoxy or carboxyl groups in the pre-polymer may form chemical interactions with functional groups on the fiber surface, further enhancing the interfacial bond strength. This reinforced interface facilitates more efficient load transfer from the resin matrix to the high-strength carbon fibers, enabling the composite to maintain high tensile strength (Figure 2: 1968 MPa) while simultaneously achieving improved toughness.

The content of EPBN plays a critical role in regulating the composite’s performance: an insufficient amount (e.g., 5%) fails to form a continuous rubber phase, resulting in limited toughening and damping effects (Figure 9: damping ratio 1.37%); conversely, excessive addition (e.g., 35%) causes the rubber phase domains to become oversized, softening the epoxy network and leading to reduced modulus and strength. At the optimal formulation (CF-EP25%), the rubber phase exhibits a well-distributed morphology with moderate domain size, maximizing energy dissipation efficiency while preserving the rigidity of the epoxy framework, thereby achieving an optimal balance among toughness, strength, and damping performance. In summary, the composite material with 25% EPBN achieves optimal performance, successfully combining high mechanical strength, excellent toughness, and outstanding damping properties. Chen Qing et al. [38] employed infrared spectroscopy to analyze the pre-polymerization and curing reactions of CTBN-toughened modified epoxy resin. The results indicate that the terminal carboxyl groups of CTBN react with the epoxy groups of EP. At a CTBN content of 25 phr, the modified epoxy resin exhibits a 33% increase in tensile shear strength, a 528% improvement in impact strength, a 70% reduction in flexural strength, and an 84% decrease in flexural modulus. These findings align with the optimal EPBN content determined in this study.

## 5. Conclusions

In this study, a novel modified epoxy resin-based carbon fiber damping composite was fabricated using a co-curing process. The following conclusions can be drawn from the investigation:

1. At 25% EPBN content, the material achieves an optimal balance between tensile, flexural, and damping properties, while its impact strength is also retained at a high level: tensile strength reaches 1968 MPa, tensile strain is 1.14%, and tensile modulus is 107 GPa. Additionally, the flexural strength reaches 1092 MPa with a flexural modulus of 105 GPa, and the impact strength peaks at 115 kJ/m^2^, all corresponding to the CF-EP25% composition;
2. Characterization by infrared spectroscopy, thermogravimetric analysis, and differential scanning calorimetry indicates that at the CF-EP25% addition level, the carboxyl-terminated butyraldehyde and epoxy resin are fully crosslinked, with no residual –OH or other unreacted functional groups detected. After blending with carbon fiber, the modified epoxy resin exhibits significantly improved mechanical properties and enhanced thermal stability;
3. Dynamic mechanical analysis indicates that as the content of carboxyl-terminated butyronitrile increases, the damping loss factor of the epoxy resin–carbon fiber composite first rises and then declines. The maximum damping ratio of 1.67% is achieved with the CF-EP25% formulation, indicating optimal damping performance. In contrast, the CF-EP5% composition exhibits a lower damping ratio of 1.37%, corresponding to weaker damping capability.

Carbon fiber-reinforced composites offer advantages such as lightweight construction, low density, high modulus-to-weight ratio, and resistance to acids and alkalis. They can withstand prolonged exposure to harsh environments and perform reliably under extreme operating conditions. In the aerospace sector, they can be used for load-bearing structural components such as aircraft fuselages and satellite mounts; in the new energy vehicle and rail transit sectors, they can be used for lightweight automotive components and high-speed train bodies; in the sports equipment sector, they can be employed in non-uniform diameter primary load-bearing components for products such as carbon fiber bicycles, tennis rackets, and gymnastics apparatus. This study has improved the material’s damping properties, impact resistance, and bending performance to some extent. This research provides foundational data for epoxy resin-based carbon fiber-reinforced composites. However, this study has certain limitations, such as the lack of systematic investigation into the long-term durability of the material under complex environmental conditions, its damping behavior at different frequencies, and the process stability for scaled-up production. Future research should focus on aspects such as interface regulation, environmental adaptability, and engineering application validation to provide a more comprehensive foundation for the practical use of such materials.

## Figures and Tables

**Table 1:** Comparison Chart of Mechanical Properties of Carbon Fiber Composites with Different EPBN Content.

Sample	Maximum Force N	Standard Deviation	Tensile Modulus GPa	Standard Deviation	Tensile Strength MPa	Standard Deviation	Tensile Strain %	Standard Deviation
CF-EP5%	54,390	450	120	4.3	1813	20	0.88	0.11
CF-EP15%	57,150	360	114	3.5	1905	23	1.03	0.09
CF-EP25%	59,040	480	107	3.4	1968	16	1.14	0.10
CF-EP35%	58,020	420	104	2.8	1934	19	1.27	0.06

**Table 2:** Radar chart of Flexural performance for carbon fiber composites with Different EPBN content.

Sample	Flexural Modulus GPa	Standard Deviation	Flexural Strength MPa	Standard Deviation	Flexural Strain %	Standard Deviation
CF-EP5%	118	5	1343	26	1.27	0.11
CF-EP15%	111	4	1229	30	1.40	0.10
CF-EP25%	105	3	1092	32	1.50	0.09
CF-EP35%	100	4	1037	36	1.70	0.12

**Table 3:** Mass Loss Table for Carbon Fiber Composites with Different EPBN Content.

	First Stage	Mass Loss %	Second Stage	Mass Loss %	Third Stage	Mass Loss %
CF-EP5%	20~361 °C	2.2	361–467	25.8	467–800	2.9
CF-EP15%	20–360	2.3	360–478	26.6	478–800	2.2
CF-EP25%	20–349	1.62	349–474	26.8	474–800	2.5
CF-EP35%	20–349	1.5	349–467	26.6	467–800	2.6

## Data Availability

The original contributions presented in the study are included in the article. Further inquiries can be directed to the corresponding authors.
